# Ratios for double silicone oil Endotamponade – in vitro observations may assist with ratio selection

**DOI:** 10.1186/s12886-017-0660-7

**Published:** 2017-12-28

**Authors:** Cheryl MacGregor, Abigail Jonas, Abdul Hanifudin, Jonathan Lochhead

**Affiliations:** 1 Ophthalmology Department, St Mary’s Hospital, Parkhurst Road, Newport, Isle of Wight PO30 5TG UK; 2 Royal Bournemouth Hospital, Bournemouth, UK; 3 Southampton University Hospital, Southampton, UK

**Keywords:** Silicone oil, Retinal detachment, Double silicone oil endotamponade, Complex retinal detachment

## Abstract

**Background:**

Silicone oil tamponade is more frequently reserved for cases of complex retinal detachment. We describe the effects of different variations in oil ratios with the relatively unknown technique of double oil tamponade.

**Methods:**

Retrospective case note review of nine patients with complex rhegmatogenous retinal detachment (RD). All cases had both superior and inferior breaks, mostly with associated proliferative vitreoretinopathy (PVR). All cases were treated with pars plana vitrectomy (PPV) and a double silicone oil endotamponade (DSOE) of both heavy silicone oil and conventional ‘light’ silicone oil. Ratios were varied to suit different RD configurations. In vitro observations were studied to help direct these decisions.

**Results:**

Anatomical success was achieved in all cases. Common complications were the same as those seen in single oil tamponade (elevated intraocular pressure, cystoid macular oedema (CMO), cataract and posterior capsule opacification. No single case of recurrent RD was seen whilst mixed oil remained in situ.

**Conclusions:**

Double silicone oil endotamponade is a safe and effective treatment for complex retinal detachments with superior and inferior breaks. Differences in oil ratios can be tailored to best fit the distribution of retinal pathology. In vitro observations may help to inform these choices.

## Background

Silicone oil tamponade is more frequently reserved for cases of complex retinal detachment (RD) [1]. Silicone oils can be divided into ‘conventional’ oils such as Arciolane (Arcadophtha, Toulouse, France) which are lighter than aqueous and are best for tamponading superior breaks, and ‘heavy’ oils such as Densiron (Fluoron, Neu Ulm, Germany) which are heavier than aqueous and effective for tamponading inferior breaks [1, 2].

Complex RDs with both superior and inferior breaks and/or proliferative vitreoretinopathy (PVR) [3] present a challenge, as neither type of oil provides adequate tamponade [4]. We report a series of such cases who received tamponade with both types of oil simultaneously to enable tamponade of both superior and inferior retina. The cases selected for DSOE were principally governed by distribution of breaks and PVR, which typically involved 3 or more quadrants thus requiring a superior and inferior tamponade at detachment repair.

This technique has been described [5] but is not widespread in UK vitreoretinal practice.

**Fig. 1:**
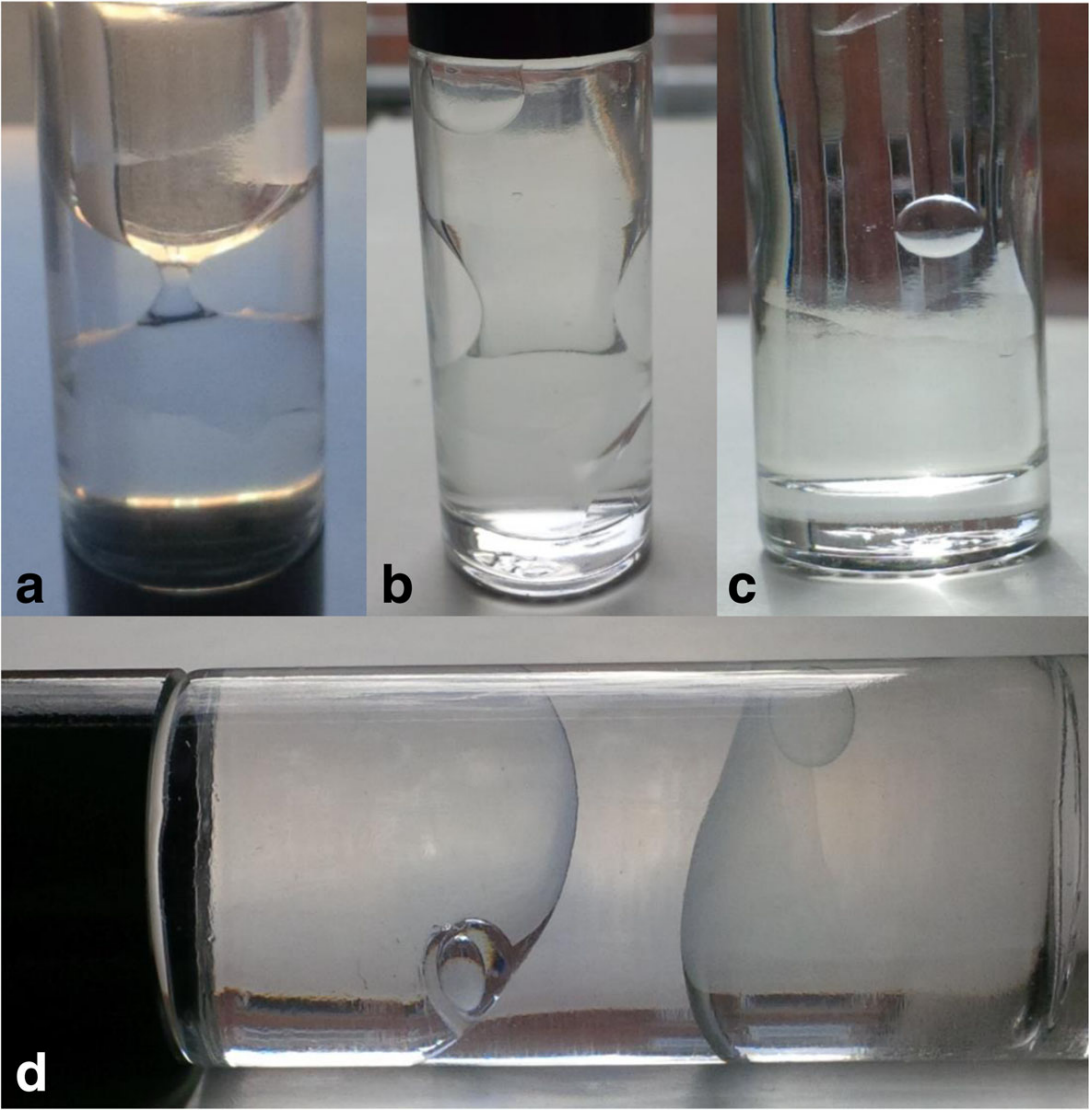
**a** DSO ‘underfill’ with minimal contact at interface. **b** Moderate ‘fill’ with Doughnut BSS at interface. **c** Optimum ‘fill’ with subtle ‘Hourglass’ contour at interface. Single bubble of BSS suspended between 2 layers. **d** Illustrates the effect of light and heavy specific gravity oil within the same container each demonstrating a clear tendency to float or sink. This tendency is further highlighted by the location of the smaller suspended BSS bubbles

The use of varied oil ratios has not been previously reported. We examined some of the in vitro configurations of these mixtures and reflect on how different oil ratios might be selected to suit different clinical presentations (Fig. 1a-d).

## Methods

We performed a retrospective case note review of 9 patients with complex macular off rhegmatogenous RD. All cases had both superior and inferior breaks, mostly with PVR. All patients were treated with 25-gauge PPV followed by double silicone oil endotamponade (DSOE). Densiron 68 (‘Heavy’ SO) was inserted first, followed by Arciolane 1300 (‘light’ SO). Ratios were varied to suit different RD configurations. Removal of silicone oil (ROSO) was performed using 25-gauge PPV and an additional 18-gauge port. In phakic eyes, cataract surgery was routinely performed at time of ROSO. In vitro observations were studied to guide clinical decision-making.

## Results

All cases are summarized in Table 1.

**Table 1:** Demographics and Results

*Case*	*Age range (Years)*	*R/L*	*Lens status*	*Macula*	*PVR Grade* 3	*Ratio of Densiron:SO*	*Duration of DSOE (months)*	*Pre-operative BCVA Snellen*	*Post-operative BCVA Snellen*	*Pre-operative BCVA Decimal*	*Post-operative BCVA Decimal*	*Complications (prior to ROSO)*
1	60-70	L	Phakic	Off	A	50:50	4	6/36	6/9	0.17	0.67	None
2	70-80	R	Phakic	Off	B	50:50	5	CF	6/36	CF	0.17	CMO, PCO
3	70-80	R	Phakic	Off	A	50:50	2	2/60	6/12	0.0	0.5	Raised IOP, Uveitis
4	40-50	R	Phakic	Off	A	70:30	6	4/60	2/60	0.07	0.03	Mild raised IOP, cataract
5	50-60	R	Phakic	Off	none	80:20	5	HM	3/60	HM	0.05	Mild raised IOP, cataract
6	80-90	R	Pseudo -phakic	Off	D1	70:30	3	HM	2/60	HM	0.03	PVR progression under oil, CMO
7	50-60	R	Phakic	Off	B	70:30	5	6/60	1/60	0.17	0.67	None
8	70-80	L	Pseudo -phakic	Off	C1	70:30	2	HM	3/60	CF	0.17	Raised IOP day 1, PVR progression under oil, CMO
9	60-70	L	Pseudo -phakic	Off	D1	30:70	10	HM	6/24	0.03	0.5	None

All cases had multiple breaks, mostly associated with PVR. The mean age was 66 years with male to female ratio 6:3. All except one patient had documented PVR. The mean decimal visual acuity (VA) was 0.05 preoperatively and 0.2 postoperatively, all but 2 patients had improved acuity. Densiron:SO ratios (DSORatio) varied from 70:30(4), 80:20(1); 50:50(3) and 30:70 (1).

The mean duration of DSOE was approximately 4 months. A single case elected for long term mixed oil whilst undergoing treatment in the fellow eye. VA remained stable at 0.6, 10 months post-oil insertion (9).

Anatomical success was achieved in all cases. Although recurrent PVR TRD later occurred in three cases (3,6,8), 2 of these following ROSO, rhegmatogenous RD was not observed. These 3 cases had a recurrence of PVR TRD in a more localised area of 1 quadrant allowing tamponade with a single oil, 2 Densiron and one Arciolane 5500. One densiron has since had successful ROSO without recurrence. 2 cases (6,8) still have SO in situ.

Raised IOP was the commonest postoperative complication (four cases) but was usually mild, only persisting following ROSO in one case with pre-existing glaucoma. CMO occurred in three cases and ERM formation in 2 cases. No cases were observed of excessive oil emulsification, exaggerated postoperative inflammation, or problems with the oil-oil interface.

Our in-vitro observations looked at different SO mixtures within a glass container mixed with small amounts of aqueous (Fig. 1). The interface changed depending on the compartment ‘fill’. An ‘underfill’ left 2 distinct layers with aqueous in the centre. A more ‘adequate fill’ allowed a stable ‘hour glass’ configuration to form at the interface. ‘Optimum fill’ created a small ‘doughnut’ ring of aqueous in the periphery of the interface, which did not change over a 36-month static observation period.

## Discussion

Our results demonstrate that DSOE is a safe and effective treatment for complex RDs with superior and inferior breaks. Redetachment may still occur in cases with aggressive PVR. Common complications are the same as those observed with single oil tamponade [6] and include raised IOP, CMO and cataract. CMO in our series occurred in eyes with severe PVR or ERM.

This technique provides effective tamponade over a larger surface area than a single oil, without the need for posturing. The oil ratio is estimated from in vitro observations. Figure 1 demonstrates the aqueous ‘hour glass’ and ‘doughnut’ shapes of BSS forming at the interface. This may be an area of reduced tamponade, therefore ratios were chosen which would offer optimum tamponade to the most significant breaks and any PVR with the patient in an upright position. The effect of nocturnal posturing is unknown and effective nocturnal posturing is difficult to achieve. We therefore selected oil ratios that would allow for maximal retinal tamponade in the upright position to allow for effective tamponade during waking hours, which represents greater than 12 h per day and most importantly, requiring little patient compliance and positioning.

DSORatios of 70:30 (4–8) were used in predominantly inferior PVR RD reflecting previously reported work [5] but also in cases where macular tamponade was strongly indicated. 50:50 (1–3) was selected in cases where superior and inferior pathology were equitable and the macular was relatively spared from PVR process. A single case was selected for 30:70 based on the need for predominantly superior tamponade and macular involvement.

Our clinical observation is that failure with oil tends to occur in the area of least tamponade usually during the post op period. With mixed oil we did not observe any failure until removal, suggesting effective all round endotamponade. 3/9 cases redetached after ROSO due to localised PVR which could then be treated using a more conventional single tamponade.

Aqueous is always present within the vitreous cavity of an oil filled eye. With DSOE it is possible to position the aqueous inside the eye where it is felt to present the least threat to anatomical success.

Other mixtures have been reported, particularly that of perfluorocarbon with SO [7].

This was an attempt to achieve simultaneous inferior and superior tamponade based on the recognition that this could be very beneficial in these complex cases. This has been reported as a successful technique but carries with it significant risks if the perfluorocarbon cannot be removed after just 2 weeks. In many PVR cases this would not be an adequate duration for tamponade. Our cases were able to tolerate mixed SO for extended periods.

## Conclusion

In summary, we feel that DSOE is a safe and effective treatment for complex RD with superior and inferior breaks. Differences in oil ratios can be tailored to best fit the distribution of retinal pathology. The mixture of oils has an enduring configuration in vitro which appears to provide effective long-term tamponade in vivo. Larger studies would be beneficial to further demonstrate DSOE effectiveness and validate the indications for different oil combinations and ratios.

## Abbreviations

BCVA: Best corrected visual acuity
CF: Counting fingers
CMO: Cystoid macular oedema
DSOE: Double silicone oil endotamponade
ERM: Epiretinal membrane
HM: Hand movements
IOP: Intraocular pressure
PCO: Posterior capsule opacification
PPV: Pars plana vitrectomy
PVR: Proliferative vitreoretinopathy
RD: Retinal detachment
ROSO: Removal of silicone oil
SO: Silicone oil
VA: Visual acuity

## References

[1] Gonvers M (1985). Temporary silicone oil tamponade in the management of retinal detachment with proliferative vitreoretinopathy. Am J Ophthalmol.

[2] Wolf S, Schön V, Meier P, Wiedemann P (2003). Silicone oil-RMN3 mixture (“heavy silicone oil”) as internal tamponade for complicated retinal detachment. Retina.

[3] Retina Society Terminology Committee (1983). The classification of retinal detachment with proliferative vitreoretinopathy. Ophthalmology.

[4] Kleinberg TT, Tzekov RT, Stein (2011). Vitreous substitutes: a comprehensive review. Surv Ophthalmol.

[5] Zenoni S, Comi N, Fontana P (2012). The combined use of heavy and light silicone oil in the treatment of complicated retinal detachment with 360° retinal breaks: Tamponade effect or filling effect?. Ann Acad Med Singap.

[6] Joussen AM, Rizzo S, Kirchhof B (2011). HSO-study group heavy silicone oil versus standard silicone oil in as vitreous tamponade in inferior PVR (HSO study): interim analysis. Acta Ophthalmol.

[7] Zenoni S, Romano R, Palmieri S (2011). Ocular tolerance and efficacy of short-term tamponade with double filling of polydimethyloxane and perfluoro-n-octane. Clin Ophthalmol.

